# Effect of humidity on egg hatchability and reproductive biology of the bamboo borer (*Dinoderus minutus* Fabricius)

**DOI:** 10.1186/2193-1801-2-9

**Published:** 2013-01-11

**Authors:** Ahmad R Norhisham, Faizah Abood, Muhamad Rita, Khalid Rehman Hakeem

**Affiliations:** 1 Department of Forest Management, Faculty of Forestry, Universiti Putra Malaysia (UPM), 43400 Serdang, Malaysia; 2 Department of Plant Protection, Faculty of Agriculture, Universiti Putra Malaysia, (UPM), 43400 Serdang, Malaysia; 3 Faculty of Forestry, Universiti Putra Malaysia, 43400 UPM Serdang, Selangor Malaysia

**Keywords:** Bostrychidae, Bamboo borer, *Dinoderus minutus*, Egg hatchability, Powderpost beetle, Reproductive capacity

## Abstract

Wood products are highly exposed to infestation by powder post beetles. *Dinoderus minutus* (bamboo borer) is a wood boring beetle that seriously damage dried bamboo and finished bamboo products. Management of *D. minutus* using pesticides showed negative effects on environment despite being very costly. By understanding influence of natural climatic conditions on their reproductive behaviour, could help us to develop a cost effective and environmental friendly strategy to cope up with this problem. In the present study, reproductive parameters and egg development of the bamboo borer were determined at 20%, 40%, 56%, 75% and 85% r.h. levels at constant temperature of 30° ± 2°C with 8 L-16D photoregime. From the results, eclosion to first instar larva was recorded at all relative humidities tested. The lowest shortest percentage of hatchability was recorded at 20% and 85% relative humidity with a mean incubation period of 4.63 ± 0.25 and 10.43 ± 0.32 days, respectively. It was noted that pre-ovipositional period decreased from 14.20 ± 0.49 to 7.20 ± 0.31 days as relative humidity increased from 20% to 75% and slightly increased to 8.00 ± 0.37 days at 85% relative humidity. We conclude that female beetles may have a particular hygropreference in oviposition as total egg production increased with increasing relative humidity.

## Introduction

Plants are always under the influence of biotic as well as abiotic stressful conditions (Anjum et al. 2010; Hakeem et al. 2012). Plant-insect interactions are quite common in nature and sometimes very useful for both species, however, when insects showed negative interactions, resulting to cause direct damages to the plants or their products, they are termed as pests. Pests are known to attack the economically important plants and their products at both pre-harvest as well as post-harvest stages and responsible for huge economic losses (Agboka et al. 2010).To cope up with this problem, normally pesticides are used. However, besides being very costly, the indiscriminate use of these pesticides has now created serious health as well as environmental problems (Madhun and Freed 1990; Pimentel 2005; Devi 2007). Repeated application of pesticides leads to loss of biodiversity (Bengtsson et al. 2005). The latest trend is to understand the nature of reproductive biology of these pests and their dependence on several environmental conditions (Garcia and Morrell 2009).

Various climatic conditions are responsible for the normal reproduction, production of eggs as well as egg hatchability. Relative humidity can affect the physiology and thus the development, longevity and oviposition of many insects. At low relative humidities, development may be retarded, for example in many pests of stored products; at high relative humidities or in saturated air (100% RH), insects or their eggs may drown or be infected more readily by pathogens (Gullan and Cranston, 2005). Different exposure of relative humidity on stored product pest, *Tribolium castaneum*, *T. confusum* (Coleoptera: Tenebrionidae) and *Oryzaephilus surinamensis* (Coleoptera: Cucujidae) showed increased insect mortality as relative humidity decreased (Jay et al. 1971). Low relative humidity can prevent embryo development and egg hatching due to loss of lubrication and cuticular softness in insect (Guarneri et al. 2002). High relative humidity contributes to population increase in stored product pest, as shown in *Callosobruchus maculatus* under laboratory conditions (Ouedraogoa et al. 1996). Female *Callosobruchus maculatus* showed higher fecundity and longer adult lifespan at high humidity. High humidity support reproductive capacity in insects as percentage of water is correlated with the amount of fat (including eggs in females), which consists of anhydrous molecules, and with the amount of cuticle, which has a lower water content than other tissues. Environmental temperature and humidity affects the developmental phase and transpiration through insect body surface (Chapman 1975; Guarneri et al. 2002). Insects must keep body water content within certain limits which is influenced by the degree of the insect cuticle permeability (Willmer, 1982; Raghu et al. 2004). Insect survival is influenced by its ability to tolerate fluctuations in body water influence by humidity (Romoser and Stoffolano, 1998).

The present study was concentrated to understand the effect of relative humidity on egg hatchability and reproductive capacity of bamboo borer *(Dinoderus minutus)*. This study would help us to develop cost effective and environmental friendly strategies to control insect pests without any health hazards.

## Materials and methods

### Insect Culture

Adult *D. minutus* were collected from infested bamboo culms in Bamboo Incubation Center and Handicraft in Simpang Pertang, Negeri Sembilan, Malaysia (2°57’25.61”N 102°18’12.69”E). Most bamboo culms infested by *D. minutus* belongs to the species *Gigantochloa scortechinii*, which is an important species of bamboo in Malaysia. Infested bamboo culms were kept in a plastic container with dimension of 30 cm × 20 cm × 25 cm kept in insect rearing room at 75% r.h. and 25 ± 3°C with 8 L-16D photoregime. Newly emerged beetles were collected and introduced into a new culture jar with cassava block using standards techniques of culture described by Abood et al. ( 2010).

### Control of Relative humidity

Different salt solutions and distilled water were used to control the relative humidity to which eggs and adult beetles were exposed. As indicated by Winston and Bates (1960), Roca and Lazzari (1994) and Dambach and Goehlen (1999), acid or inorganic (non-volatile) salt solutions were adequate for this purpose, as their water vapour pressure remained constant for long periods. The liquids used to establish the relative humidities were distilled water, saturated solutions of Potassium acetae (CH_3_CO_2_K), Sodium iodide (NaI), Sodium bromide (NaBr), Sodium chloride (NaCl) and Potassium chloride (KCl). The relative humidity was obtained by filling solutions at the bottom of desiccators and monitored using a digital thermohygrometer. Throughout the study, investigation on reproductive parameters and egg hatchability were carried out in Petri dishes kept in desiccators corresponding to each relative humidity and hermetically closed. The desiccators were put into an incubator with controlled temperature of 30 ± 2°C.

### Reproductive capacity

Newly emerged beetles less than 7 days old were used to determine the effect of different relative humidities (20%, 40%, 56%, 75% and 85% r.h.) on reproductive parameters at constant temperature of 30° ± 2°C with 8 L-16D photoregime. Ten pairs of adult beetles were exposed at each relative humidity tested. Each pair of adults were selected and separately introduced into Petri dishes lined with cassava pellet and filter paper. Observation on egg production was then started after 24 hours of setup. Cassava pellets were removed and broken up daily to assess number of eggs laid. The cassava pellet was replaced each time after inspection on egg production. Data collection on adult longevity and fecundity were collected until complete mortality.

### Egg hatchability

The effect of relative humidity on egg hatchability and incubation period was inspected under different relative humidity levels (20%, 40%, 56%, 75% and 85% r.h.) at constant temperature of 30° ± 2°C with 8 L-16D photoregime. Newly laid eggs of *D. minutus* were obtained from an established *D. minutus* culture on cassava. A total of 50 eggs were placed equidistant into Petri dishes lined with black filter paper for each relative humidity tested. Each Petri dish was kept in desiccators corresponding to each relative humidity and hermetically closed. The desiccators were put into an incubator with controlled temperature of 30 ± 2°C. Petri dishes were kept under these conditions and observation on egg eclosion and incubation period were observed daily under stereomicroscope and compared. During observation, eggs were removed as required with respect to age in days with a fine brush. Each egg was mounted on a concave microscope slide with a cover slip. This method was carried out to minimize the effect of relative humidity during the non-test conditions.

### Statistical analysis

Statistical analysis software (SAS version 9.2) was used for the analysis on egg hatching, reproductive capacity and adult longevity at different relative humidities. One Way Analysis of Variance (ANOVA) and Fisher’s least significant difference (LSD) were used to test the effect of relative humidity on reproductive parameter and incubation period.

## Results

### Effect of relative humidity on egg hatchability

The results presented in Table 1 shows a very high significant effect (F=242.30, DF= 4, 45; p<0.0001) of relative humidity on egg hatchability in *D. minutus*. There is no significant difference between incubation period at 40%, 56% and 75% relative humidity (p≥0.05; CV=16.63). The lowest and highest relative humidity caused a significant decrease in egg hatchability when compared with the intermediate relative humidity levels (p≥0.05; CV=16.63).

**Table 1:** **Influence of relative humidity on egg eclosion**

Relative humidity	Egg development	
	Incubation period (day)	Hatchability
20%	4.63±0.25c	8%
40%	5.79±0.13b	66%
56%	5.49±0.18b	74%
75%	5.44±0.14b	86%
85%	10.43±0.32a	48%

Means followed by the same letter in the same column are not significantly different (p≥0.05, Fisher’s least significant difference) N=50.

Highest eclosion to first instar larva was recorded at 75% relative humidity with 86% eclosion to success and a mean incubation period of 5.44 ± 0.14 days. Lowest relative humidity of 20% showed only 8% eclosion to success with a mean incubation period of 4.63 ± 0.25. Percentage of successful eclosion and incubation period increased with increasing relative humidity to 75%. However for 85% relative humidity, the percentage of successful eclosion decreased to 46%. This shows that egg development was affected by extremes in humidity (Plate 1). Longest incubation period was recorded at 85% relative humidity with a mean of 10.43 ± 0.32 days.

### Effect of relative humidity on reproductive capacity

Boring activity on cassava pellet was recorded within 24 hours of exposure under each relative humidity. Results in Table 2 show highly significant relative humidity effect on both *D. minutus* reproductive capacity (F=388.91, DF=4, 45; p<0.0001) and adult lifespan (F=464.70, DF=4, 45; p<0.0001). However, there is no significant difference in pre-ovipositional period between 56% and 75% relative humidity levels (p≥0.05; CV; 13.14). Female *D. minutus* showed shorter pre-ovipositional period with increasing relative humidity until 75%. Ovipositional period (p≥0.05; CV=8.67) and mean number of eggs laid (p≥0.05; CV=13.14) showed no significant difference between 75% and 85% relative humidity levels. Shortest ovipositional period was recorded at 20% relative humidity with a mean of 3.8 ± 0.64 days while the longest ovipositional period was recorded at 85% relative humidity with a mean of 56.50 ± 1.21. Mean number of eggs laid increased from 3.20 ± 0.31 to 56.50 ± 1.21 from 20% to 85% relative humidity, thus indicating a preference for higher relative humidity conditions. Similar increment was recorded on mean ovipositional period which showed an increase from 3.8 ± 0.64 to 54.80 ± 0.94 days. The pre-ovipositional period decreased with increasing relative humidity levels from 20% to 75%. However, between 75% to 85% relative humidity, there was a significant increase in pre-ovipositional period. Adult lifespan for both sexes increase with increasing relative humidity. Female *D. minutus* had longer lifespan compared to male beetles under all relative humidities tested. There was no significant difference between adult lifespan for male (p>0.05; CV=7.26) and female (p>0.05; CV=5.19) between 85% and 75% and 75% and 56% relative humidity, respectively. Earliest mortality for adult beetle was recorded at 20% relative humidity for male and for female beetle on day 16 and 18, respectively. Longest lifespan for male beetle was recorded at 67 day at 75% relative humidity while longest lifespan for female beetle was 74 days at 85% relative humidity.

**Table 2:** **Effect of relative humidity on reproductive capacity**

Relative humidity	Reproductive parameter				
	Preovipositional period (day)	Ovipositional period (day)	Mean no. of eggs laid per female	Female Lifespan (day)	Male Lifespan (day)
20%	14.20±0.49a	3.80±0.64d	3.20±0.31d	22.30±0.79c	20.00±0.62c
40%	11.80±0.44b	37.20±1.34c	28.90±1.01c	56.90±1.20b	49.70±1.15b
56%	9.60±0.38c	51.80±0.49b	43.00±1.65b	68.80±0.68a	61.50±1.29a
75%	7.20±0.31c	54.40±1.33a	66.40±1.21a	70.10±0.70a	60.50±1.19a
85%	8.00±0.37d	56.50±1.21a	68.90±3.07a	69.40±1.52a	59.80±1.13a

Means followed by the same letter in the same column are not significantly different (p≥0.05, Fisher’s least significant difference) N=20.

**Figure 1:**
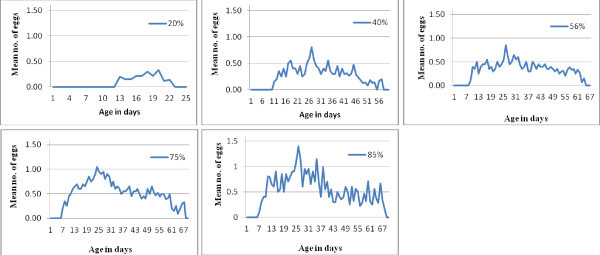
**Age-specific fecundity curves of*D. minutus*at different relative humidities.**

Fecundity curves for *D. minutus* under different relative humidity are shown in Figure 1. Fecundity curve for 40%, 56% and 75% show normal distribution with few fluctuations throughout the oviposition period. The lowest and highest relative humidity resulted in different fecundity curve pattern compared with the intermediate relative humidity. Peak fertility period was encountered earlier at 75% relative humidity on day 24 with a mean of 1.04 followed by day 27 at 56% and 85% relative humidity with a mean of 0.85 and 1.40, respectively. Peak fertility period at lower relative humidity occurred at a mean of 0.80 on day 27 at 40% relative humidity while at 20% relative humidity fecundity curve obtained did not present peak fertility period as reproductive parameters recorded under this condition was very low.

## Discussion

In order to establish a sustainable pest management, it is essential to know the physiology as well as reproductive capacity of pests viz-a-viz their relations with various natural climatic conditions. Thus present study was undertaken to investigate the effect of humidity on egg hatchability and reproductive biology of the bamboo borer (*Dinoderus minutus* Fabricus).

**Figure 2:**
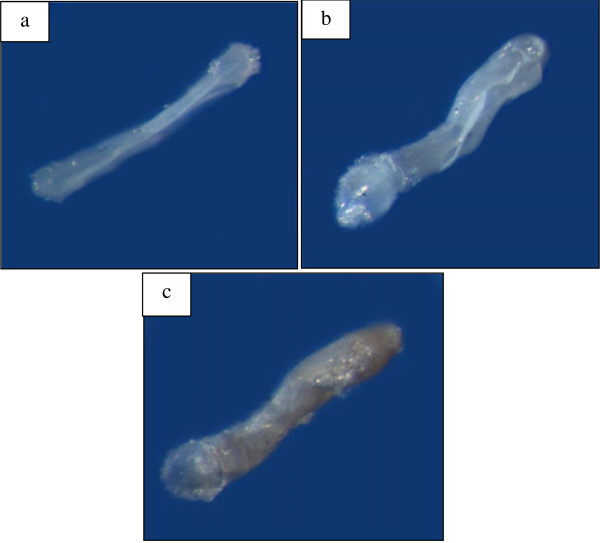
**Effects of different relative humidities on*D. minutus*egg (x40): a. Newly laid egg; b. shrinkage of egg at 20% relative humidity; c. cuticular softening due to absorption of moisture at 56% relative humidity and d. swelling of prelarva within chorion at 85% relative humidity.**

In our study, we found that egg mortality was highest at 20% and 85% relative humidity levels in *Dinoderus minutus*. Dehydration occurred at low relative humidity due to loss of moisture in the egg, which leads to contraction and shrinking of both the chorion and the embryo. For eggs, which survived the low relative humidity levels up to the prelarval stage, the eggs did hatch but due to dehydration and loss of lubrication and cuticular softness, the prelarva could not be released successfully from the chorion (Figure 2). This effect on eggs caused by loss of water has been reported by Woods and Singer (2001) on Lepidoptera. Excessive moisture has shown to be detrimental to insect’s survival (Prakash, 2008). At 85% relative humidity, the incubation period prolonged and 52% of the total number of egg exposed failed to hatch due to excessive moisture, which caused egg mortality. Previously, such negative effect of extreme relative humidity conditions on egg development and hatchability has been reported on the stink bug, *Nezara viridula* (Linnaeus) (Hirose et al. 2006) and the haematophagous bug *Triatoma brasiliensis* (Guarneri et al. 2002). The extension of embryonic development duration at lower humidities (and constant temperature) is a result of the depression of egg metabolism due to water loss (Zrubek and Woods 2006); low humidities induce in some insect eggs (collembolans, grasshoppers) a dormancy, which may last several months (Wigglesworth 1972). Similar Study by Alex (1983) on the humidity effect on *Atherigona soccata* showed that low humidity increases water loss on the chorion, and resistance to desiccation depends on the ability of the egg to retain water through physical and physiological processes.

From the observations in this study, most eclosion to first instar larva of *D. minutus* took place in the morning. During this period, relative humidity reaches its daily maximum. This may be a way to prevent desiccation of emerging larvae. This phenomenan of insect egg hatching at dawn has also been reported previously in different heteropteran species (Lazzari, 1991; Guarneri et al. 2002) and the tiger moth, *Atteve sciodoxa* Meyrick (Bajwa, unpubl. data 2009).

Female *D. minutus* showed heterogeneous ovipositional pattern with most eggs being laid at higher relative humidities and the mean number of eggs laid increased from 3.20 ± 0.31 to 56.50 ± 1.21 at 20% to 85% relative humidities, respectively. The effect of relative humidity on oviposition indicates that females may have a particular hygropreference in oviposition as total egg produced and ovipositional period increased with increasing relative humidity. However, observation on egg hatchability showed that high relative humidity of 85% resulted with low egg hatchability (Table 1). These results might explain how female *D. minutus* was capable to reproduce at high relative humidity at early infestation in bamboo storage. As relative humidity decreased during the processing of bamboo culm, egg hatchability increases causing a pest outbreak. Romoser and Stoffolano (1998) stated that low humidity adversely affect the rate of insect oviposition in general. A study by Saha et al. ( 2011) showed that total progeny of *Xylocoris flavipes* decreased at high relative humidity of 90%. David and Ananthakrishnan (2004) suggested that the fecundity of an insect is greatest at 70% relative humidity, as seen in *Locusta* spp. Andrewartha and Birch (1954) reported that newly emerged adult migratory locusts did not produce eggs below 40% relative humidity. *D. minutus* response towards relative humidity was found to be similar to the rice weevil, *Sitophylus oryzae,* an important pest in stored product*.* Fecundity of *S. oryzae* increased with increasing moisture content of wheat from 34% to 70% relative humidity (David and Ananthakrishnan, 2004).

## Conclusion

The effect of relative humidity on *D. minutus* egg hatchability showed high mortality at 20% and 85% relative humidity levels compared to that between 40% to 75%. Lowest egg hatchability was recorded at 20% and 85% relative humidity levels. *D. minutus* egg suffers from loss of moisture that leads to shrinkage of both the embryo and chorion which prevents larval release. High relative humidity at 85% resulted in egg mortality due to excessive moisture. Reproductive capacity and incubation period of *D. minutus* increased with increasing relative humidity. Mean number of eggs laid increased from 3.20 ± 0.31 to 56.50 ± 1.21 from 20% to 85% relative humidity. Female beetle preferred to lay eggs in higher relative humidity as the number of eggs increased with increasing relative humidity. Pre-ovipositional decreased with increasing relative humidity. However at 85% relative humidity, pre-ovipositional period increased slightly compared to 75% relative humidity. Ovipositional period prolonged as relative humidity increased. Adult female showed higher fecundity characteristics with increasing relative humidity. Mean ovipositional period increased from 3.8 ± 0.64 to 54.80 ± 0.94 days with increasing relative humidity. The present study would help scientist to work out the strategy/s about the biological control of pests without causing any damage to health, environment as well as economy.
